# The evaluation of changes in peripheral neuropathy and quality-of-life using low-frequency electrostimulation in patients treated with chemotherapy for breast cancer: a study protocol

**DOI:** 10.1186/s13063-018-2874-2

**Published:** 2018-09-29

**Authors:** Chang eun Jang, Mi Sook Jung, Eun Hee Sohn, Mijung Kim, Hwa-Seung Yoo, Kyeore Bae, Je Ryong Kim, Jin Sun Lee

**Affiliations:** 1 Department of Surgery, Yonsei University College of Medicine, Seoul, Korea; 2 College of Nursing, Chungnam National University, Daejeon, Korea; 3 Department of Neurology, Chungnam National University Hospital, Daejeon, Korea; 4 East-West Cancer Center, Dunsan Korean Medicine Hospital of Daejeon University, Daejeon, Korea; 5 Departmetn of Surgery and Research Institute for Medicinal Sciences, College of Medicine, Chungnam National University, 33 Munwha-ro, Jung-gu, Daejeon, Korea

**Keywords:** Chemotherapy-induced peripheral neuropathy, Health-related quality of life, Low-frequency electrostimulation device, Breast cancer

## Abstract

**Background:**

Chemotherapy-induced peripheral neuropathy (CIPN) is a progressive, enduring, and sometimes irreversible neurotoxic symptom that occurs in 30–40% of chemotherapy-treated cancer patients. CIPN negatively affects both the patient’s abilities to perform daily activities and their health-related quality of life (HRQOL) after chemotherapy treatment. Although this neuropathy has been treated with duloxetine and/or gabapentin, limited therapeutic benefits have been reported, thereby necessitating the development of an integrated approach that combines pharmacological management and complementary methods such as acupuncture and electric nerve stimulation. Therefore, this study is designed to examine the effect of a portable, low-frequency electrostimulation (ES) device on CIPN symptoms and HRQOL of female patients diagnosed with CIPN immediately after chemotherapy for breast cancer.

**Methods:**

This study is a single-center, randomized, placebo-controlled trial with two parallel groups and a 2-week follow-up. We will enroll 80 breast cancer patients who are newly diagnosed with CIPN after chemotherapy. Duloxetine or pregabalin will be prescribed to all participants from the initial assessment. Half of the patients will be assigned into the experimental group and the other half to the control group. The CarebandR (Piomed Inc., Seoul, Korea), a wearable wristband that generates low-frequency electrostimulation, will be administered only to the experimental group. Electrostimulation will be administered on the unilateral PC6 acupoint. A numerical rating scale will be used to assess the overall intensity of CIPN symptoms. The key secondary outcome variables include patient-reported CIPN symptom distress tested by a self-rated questionnaire, physician-rated symptom severity assessed by the Total Neuropathy Score, and HRQOL.

**Discussion:**

It is expected that the combination of a low-frequency electrostimulation device and pharmacological intervention (duloxetine or pregabalin) will produce synergistic effects in breast cancer patients with CIPN after treatment. To our knowledge, this is the first study to investigate the beneficial effect of a new integrated approach for CIPN management after breast cancer treatment. The study findings can expand our knowledge and understanding of the occurrence of CIPN and the efficacy of integrated intervention efforts to ameliorate CIPN symptoms.

**Trial registration:**

Clinical Research Information Service (CRIS), Republic of Korea, ID: KCT0002357. Registered retrospectively on 13 June 2017*.*

**Electronic supplementary material:**

The online version of this article (10.1186/s13063-018-2874-2) contains supplementary material, which is available to authorized users.

## Background

According to the latest national cancer statistics, the prevalence rate of breast cancer in Korea has increased more than three times in the past 10 years and the 5-year cancer survival rates have increased from 77.9% in 1993–1998 to 91.5% in 2008–2013 [1, 2]. It is noteworthy that the number of premenopausal breast cancer patients in Korea is significantly higher than those in Western countries, which can be attributed to the fact that the highest prevalence rate is found in women aged 40 to 49 years [3]. Women diagnosed with breast cancer at age 50 years or younger reported to have poorer health-related quality of life (HRQOL) than their older counterparts because they may face various HRQOL issues for the entire trajectory of their cancer diagnosis and treatment [4]. HRQOL has been perceived as an important clinical outcome for long-term survivorship among women living longer with cancer. For this reason, the balance between survival advantages from curative therapy and HRQOL challenges has received special attention from healthcare professionals as well as breast cancer patients.

Anticancer treatment, especially chemotherapy, was associated with acute or late toxicities which affected post-treatment HRQOL after treatment [5, 6].

Chemotherapy-induced peripheral neuropathy (CIPN) is a commonly reported symptom affecting HRQOL in chemotherapy-treated breast cancer patients [7, 8]. This is referred to as any damage, inflammation, or degeneration of the peripheral nerves because of the administration of certain chemotherapeutic agents such as paclitaxel, docetaxel, or vincristine [9]. CIPN manifests as sensory, motor, and autonomic symptoms. Sensory neuropathy is the most common but motor symptoms and autonomic dysfunctions may occur [10]. Symptoms of sensory neuropathy include numbness, tingling, burning of hand and feet, loss of sensation, paresthesiae, and occasionally pain. Motor symptoms present as impaired motor nerves, weakness of muscles, and muscle atrophy. Symptoms associated with autonomic nervous system dysfunction include constipation, orthostatic hypotension, and urinary incontinence [11]. The neuropathy symptoms affect the patient’s quality of life and can cause long-lasting complications [11].

Clinically, CIPN has been treated with duloxetine and/or gabapentin. Duloxetine is a serotonin and norepinephrine dual reuptake inhibitor that can reduce pain due to the suppression of painful peripheral stimuli transmission [7]. Gabapentin can inhibit neuronal activity and thus produces analgesic effects through the reduction of excitatory neurotransmitter release [12]. The American Society of Clinical Oncology guidelines suggest that these agents are useful in managing CIPN during and after chemotherapy [13]. However, many studies have demonstrated mixed results on the efficacy of pharmacological interventions on CIPN in individuals treated with chemotherapy for various types of cancer such as breast and colorectal cancer. Interestingly, one phase 3 randomized crossover trial reported that there was no significant change in CIPN symptoms between patients treated with 2700 mg of gabapentin for CIPN and disease- and treatment-matched controls who received placebo treatment [14]. Moore et al. reported that about 74% of patients who have painful neuropathic pain can expect neuropathic pain control [15]. Due to the limited therapeutic benefits of medication for CIPN treatment, a number of studies suggest that complementary methods can be used for the treatment of CIPN in cancer patients and survivors [16, 17]. These studies have shown that various types of acupuncture, including manual acupuncture, electroacupuncture, and auricular acupuncture, have positive effects on pain symptoms in patients with cancer [16, 18]. In other studies, acupuncture-like transcutaneous electric nerve stimulation (TENS) also produced similar effects on sensory and motor symptoms of CIPN through a possible mechanism of top-down pain regulation of the central nervous system [19, 20]. Previous experimental studies have demonstrated that the analgesic effects of electroacupuncture on neuropathic pain were produced by antinociception, which involves μ, δ, or κ opioid receptors, spinal serotonin, and norepinephrine, and changes in the concentrations of excitatory and inhibitory amino acid neurotransmitters to stop the pain. The actions of electroacupuncture were also associated with glial cells and cytokines and other bioactive molecules [21]. Park et al. [22] reported that CIPN-induced pain was reduced by using electroacupuncture in patients who were treated with eight cycles of chemotherapy, including docetaxel and taxane agents. Smith et al. reported that an electrocutaneous nerve stimulation device (MC5-A Calmare) reduces CIPN pain by 20% [20]. Taken together, pharmacological treatment yields clinically beneficial effects on CIPN management, but the combination of other supportive interventions with current conventional treatments is required to complement the limited efficacy of using medication alone. Moreover, the aforementioned supportive interventions require patients to be in a fixed position for a certain period of time. To ensure that patients comply with the treatment for CIPN, the development of interventions that allow patients to work while receiving treatment anywhere and anytime is crucial.

Therefore, the purpose of this study is to examine the effect of a portable, low-frequency electrostimulation device on CIPN symptoms and HRQOL of female patients diagnosed with CIPN immediately after chemotherapy for breast cancer.

## Methods/design

**Fig. 1:**
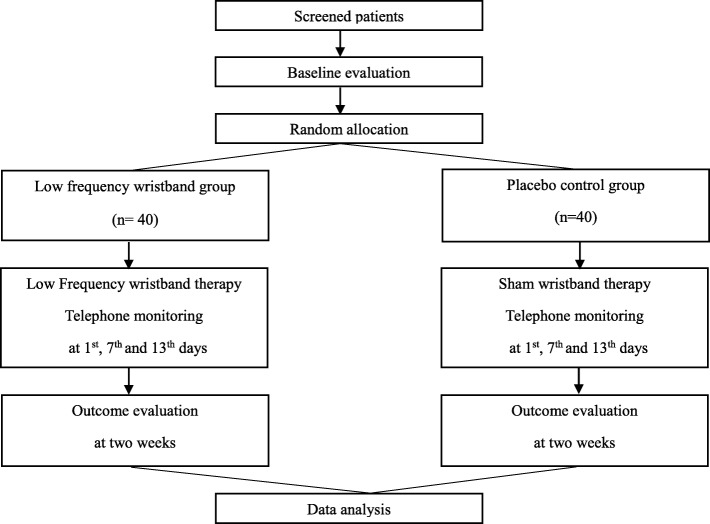
Flow chart of the trial

**Fig. 2:**
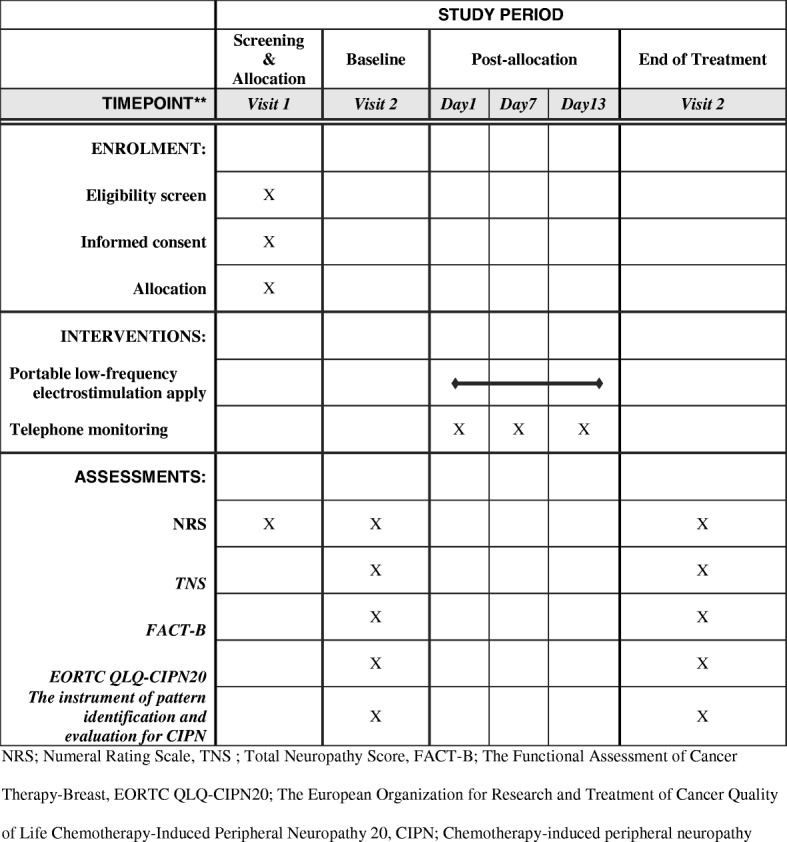
Measures

This study is a single-center, randomized, placebo-controlled trial with two groups and a 2-week follow-up. The treatment period was determined based on previous studies [20, 22–27]. The trial will be conducted at the Chungnam National University Hospital Cancer Center from March 2017 to December 2018. Trained researchers will enroll 80 breast cancer patients who are newly diagnosed with chemotherapy-induced peripheral neuropathy. Patients who meet the eligibility criteria will be recruited and randomly allocated into intervention or control groups in a 1:1 ratio. The overall flow of the trial is shown in Fig. 1. The protocol of this study has been reviewed and approved by the Institutional Review Board of Human Research of the Chungnam National University Hospital (2016–11-020). Informed consent will be obtained from all individual participants before starting any data collection in this study. The protocol fulfills the populated Standard Protocol Items: Recommendations for Interventional Trials (SPIRIT) Check list and Figure (as Fig. 2) and the SPIRIT Checklist is attached as Additional file 1.

### Participants

Participants will be considered for enrollment if they meet the eligibility criteria and have a score of at least 5 on a numeral rating scale(NRS) used to assess overall intensity of CIPN symptoms after completion of chemotherapy for primary breast cancer [28]. Inclusion criteria will be determined by one oncologist and one neurologist. Also, severe CIPN will be determined by the neurologist. More detailed eligibility information on the eligibility criteria is presented in Table 1.

**Table 1:** Patient eligibility criteria

Inclusion criteria	Exclusion criteria
Female, at least 19 years old	Patients with a history of receiving chemotherapy for cancer before this diagnosis was made
Patients diagnosed with primary breast cancer	Patients with symptoms of peripheral neuropathy that are severe and need to be immediately treated with surgical procedures or acute management
Patients with a diagnosis of CIPN presenting within the first week of an average pain score of at least 5 on a visual analogue scale of pain after completion of chemotherapy	Patients with pre-existing peripheral neuropathy due to trauma or intercurrent illness
Patients who do not take any medication to prevent or treat neuropathy before screening	Patients with skin inflammation at the attachment site
Patients with an ECOG performance status of 2 or less	Patients with a history of previous use of an acupressure wristband over a week. Patients with a history of cardiovascular disorders including the use of a pacemaker
Patients who are willing and able to comply with the requirements of the study	Patients with known hypersensitivity to metal or any medication
Patients who are willing and able to provide written informed consent	Patients with uncontrolled hypertension (systolic blood pressure ≥ 170 mmHg or diastolic blood pressure ≥ 100 mmHg) or uncontrolled diabetes
	Patients with hepatic insufficiency or dysfunction (three times higher than normal levels of ALT, AST, creatinine)
	Patients who were pregnant or are potentially pregnant and breastfeeding
	Patients with psychiatric disorders or who take medication for psychiatric illnesses
	Patients with a history of receiving investigative, new drugs within 4 weeks before enrollment
	Patients who do not have the ability to read, understand, or respond to questionnaires
	Patients with other reasons not to comply with this study protocol

*CIPN* chemotherapy-induced peripheral neuropathy, *ECOG* Eastern Cooperative Oncology Group, *AST* aspartate transaminase, *ALT* alanine aminotransferase

### Sample size

Sample size will be decided by the primary outcome. According to the published study on low-frequency electroacupuncture treatment for CIPN, the overall change in mean scores between baseline and 2-week follow-up was − 4.0 with a significant decrease in pain over time [27, 29]. However, there was no previous study that could provide data to compute standardized differences between two means of two groups in calculating the sample size for this trial. Accordingly, this study will evaluate the efficacy and feasibility of the low-frequency wristband intervention for patients with CIPN [30]. With consideration for the maximum number of subjects needed in pilot studies and the central limit theorem, a total sample size of 40 per group was calculated [31]. The sample size was calculated based on the changes in the NRS between baseline (visit 2) and the end of the study (visit 3). Based on two previous studies [26, 29], the mean and standard deviation are conservatively assumed. We calculated that 32 patients per group would be required to achieve a power of 95% with a two-sided significance level of *p < 0.05* for detecting the superiority of treatment with Careband R by a *t* test. Here, the assumed effect size (*d*) was 2.33. To account for possible dropouts (10%), the target number of patients was, therefore, set at 40 per group (80 in total) (see below):

$$ {\displaystyle \begin{array}{l}\mathrm{n}=2\kern1em {\left\{\frac{\left({\mathrm{Z}}_{1-\upalpha /2}+{\mathrm{Z}}_{1-\upbeta}\right)\kern0.5em \ast \kern0.5em \upsigma}{\varDelta}\right\}}^2\\ {}\mathrm{n}=2\kern1em {\left\{\frac{\left(1.96+1.64\right)\kern0.5em \ast \kern0.5em 2.57}{2.33}\right\}}^2\\ {}\mathrm{n}=31.63\end{array}} $$

*H*_*0*_: *d*_*t*_ *= d*_*c;*_ *H*_*1*_: *d*_*t*_ *≠ d*_*c*_*;* d_*t*_: the changes in the NRS between baseline (visit 2) and the end of the study (visit 3) of the intervention group; *d*_*c*_: the changes in the NRS between baseline (visit 2) and the end of the study (visit 3) of the control group; *Δ*; *d*_*t*_ − *d*_*c*_.

### Randomization

A total of 80 participants who meet the eligibility criteria will be randomly assigned in a ratio of 1:1 to the intervention or the placebo control group. The concomitant use of neuropathy medications during the study is a confounding factor that can influence the outcomes of the study. For this reason, the randomization will be stratified according to the use of medication administered for neuropathy. A randomization code list was generated using SAS version V9.4 (SAS Institute Inc., Cary, NC, USA) by a statistician who is not involved in this study. The randomization procedure will be conducted by a research assistant who will not take part in the interview, data collection, intervention, or statistical analysis. Obtaining informed consent and baseline data will be completed before randomization. A link between the randomization code and the corresponding treatment will remain blinded for all other members in the study team.

### Recruitment strategies

Potential candidates will be invited by a physician in the study team and will be given the details of this clinical trial. A secondary recruiting strategy, such as advertisements in the cancer center, will also be applied at the same time. Participants who meet the eligibility criteria and are willing to participate will be enrolled after they read and sign an informed consent form.

### Intervention

At baseline, the interventional medical devices and placebo medical devices will distributed to the participants allocated in the electrostimulation (ES) group and the placebo control group, respectively. The device that is used to manage CIPN symptoms is CarebandR (Piomed Inc., Seoul, Korea), which is a wearable wristband that generates low-frequency electrostimulation. The placebo wristband device, which does not generate an electrical stimulus and has an identical appearance, will be administered to the placebo control group. Both interventional and placebo devices are labeled identically for blinding of participants. The participants will be educated to use the devices on the second stage (100 μA, 40 Hz) or above for 14 days, at least twice a day, for at least 120 min, which includes one consecutive hour of use. Electrostimulation will be administered on the unilateral PC6 acupoint, which is located approximately three finger breadths above the wrist crease between the palmaris longus tendon and flexor carpi radialis tendon [32]. The PC6 acupoint was selected for its applicable location and therapeutic effects of regulating *qi*, tranquilizing, and relieving pain.

### Data collection methods and record-keeping

Data obtained from all participants will be recorded in the case report form. Researchers who are involved in data collection will be trained to ensure standardized procedures and guidelines for data collection. All data will be stored in a locked cabinet and then coded using a unique numerical identification system. The data will be entered into an electronic database by the researchers who will receive permission to access the database for this study. Logical checks will be performed to find missing data and inconsistencies.

### Measurements of outcomes

Measures are presented in Fig. 2.

### Primary outcome

The primary outcome measure of this study is the overall intensity of CIPN symptoms in women treated with adjuvant chemotherapy for breast cancer. A NRS will be used to assess the overall intensity of CIPN symptoms. This scale is the most widely used clinical tool and its validity has been assessed in studies on patients with cancer [33, 34]. Patients will be asked to rate their symptom intensity on an 11-point scale ranging from 0 (no symptoms) to 10 (worst possible symptoms).

### Secondary outcomes

The key secondary outcome variables include physician-rated symptom severity assessed by the Total Neuropathy Score (TNS), patient-reported CIPN symptom distress level tested with a self-rated questionnaire and, and perceived HRQOL, and the instrument for pattern identification and evaluation for CIPN.

The TNS will be used to assess the presence, severity, and location of symptoms. This scale was originally developed to evaluate diabetic neuropathy and later validated in cancer patients with peripheral neuropathy [35, 36]. The TNS can provide combined information obtained from pin-prick, vibration threshold, and nerve conduction studies. The TNS (range 0 to 40) combines 10 items of symptom scores, including sensory, motor, autonomic symptoms, pin sensibility, vibration sensibility, and deep tendon reflexes [37, 38]. Each neuropathy item will be scored by a neurologist on a 0 to 4 scale. The reliability and validity of this scale were determined in various studies in patients with CIPN [36, 37, 39].

The Functional Assessment of Cancer Therapy-Breast (FACT-B) will be administered to measure the primary domains of wellbeing (i.e., physical, social/family, emotional, and functional wellbeing) and a breast cancer-specific subscale that assesses symptoms and concerns related to breast cancer [40]. The FACT-B is a 37-item measure and has demonstrated good reliability, validity, sensitivity, and suitability for use in various international studies [41–43].

The European Organization for Research and Treatment of Cancer Quality of Life (EORTC-QLQ) Chemotherapy-Induced Peripheral Neuropathy 20 (CIPN20) will be used to evaluate patients’ experience of symptoms and functional limitations related to CIPN. This instrument consists of three subscales: (1) sensory (nine items), (2) motor (eight items), and (3) autonomic (three items) symptoms and functioning. Each item is rated on a 4-point Likert scale ranging from 1 (not at all) to 4 (very much) [8]. The CIPN20 was established as a reliable and valid measurement tool for individuals with CIPN [44, 45].

The instrument of pattern identification and evaluation for CIPN will be used to diagnose and evaluate CIPN based on the perspective of traditional oriental medicine [46]. This instrument includes 30 items, which can be divided into four patterns such as wind arthralgia, cold arthralgia, dampness arthralgia, and arthralgia of the deficiency type. All items are scored by an oriental medicine physician on a 5-point Likert scale and higher scores indicate greater distress in each CIPN pattern.

### Safety and adverse events

Patients will be interviewed at each visit for assessment and asked about the occurrence of any adverse events. All adverse events related to this clinical trial will be recorded and presented in the case report form in detail. The study investigator will report serious adverse events, including death, a life-threatening event, inpatient hospitalization or prolongation of existing hospitalization, and persistent or significant disability, to the Institutional Review Board of Human Research of the Chungnam National University Hospital. In addition, a safety analysis will be performed to identify the incidence of adverse events, serious adverse events, and the number and percentage of patients reporting at least one adverse event in each group.

### Withdrawals

Participants will be free to withdraw from the study at their own request at any time without giving reasons for their decision. Withdrawals will be documented in the case report forms and an active follow-up will be conducted to monitor the occurrence of serious adverse events throughout the entire planned period of study.

### Data analysis

Statistical analysis will be performed using SPSS version 22 (IBM SPSS Inc., Chicago, IL, USA) software by a statistician blinded to patient allocation. The efficacy of the intervention will be analyzed using a full analysis set approach with the last-observation-carried-forward method for primary outcome. Demographic and clinical characteristics will be described as frequency and composition ratio. Categorical data will be described as frequency and percentage. Continuous data will be presented by mean and standard deviation or median and interquartile range according to whether the data are normally distributed. For comparison with the baseline outcomes between groups, continuous data will be analyzed by using independent *t* tests or Wilcoxon rank sum tests and categorical data will be analyzed by the chi-squared or Fisher’s exact test. Mean differences from baseline to post treatment in each group will be calculated to evaluate the efficacy of treatment. Analysis of covariance will be used to analyze a difference in primary and secondary outcomes between groups if the residuals are normally distributed, otherwise the Wilcoxon rank sum test will be conducted. Clinically relevant covariates will be included in these analyses. A comparison between responders and non-responders will be conducted to explore heterogeneity of any key variables. A *p* level of 0.05 (two-tailed test) will be considered to indicate statistical significance for all analyses.

## Discussion

CIPN is a chemotherapy-induced complication that is underestimated by healthcare professionals and even patients themselves, possibly due to a lack of concern or awareness. Scholarly attention has been paid and better understanding has been established with the publication of empirical findings about the impact of chemotherapy-related symptom management on quality of life [47, 48]. However, no promising interventions for CIPN prevention or treatment exist to date.

The prevalence rate of peripheral neuropathy has increased, ranging from 19 to 85% in chemotherapy-treated cancer patients. Seretny et al. reported the pattern of CIPN incidence during and after chemotherapy. Specifically, this type of peripheral neuropathy emerged after the initiation of chemotherapy in 1960 (46.9%) out of 4179 cancer patients and was observed in more than half of patients, with 68.1% showing it immediately after completion of chemotherapy and 60% 3 months after chemotherapy. Although the rate of CIPN decreased after 3 months, 30% of patients still suffered from CIPN symptoms 6 months after chemotherapy [10]. This result showed that CIPN is a persistent problem that can act as a risk factor for an impaired quality of life after chemotherapy.

It is well known that CIPN might be caused by particular types of chemotherapeutic agents including microtubule-targeting agents, mainly vinca alkaloids and taxanes [11, 49]. According to recently published studies, epothilone and ixabepilone are also associated with the occurrence of CIPN after chemotherapy [11, 49]. Of these agents, 5-fluorouracil, etoposide, gemcitabine, and taxane need to be acknowledged as important agents in patients with breast cancer because these agents are known to be associated with CIPN after breast cancer treatment [50, 51]. Although CIPN does not directly affect overall survival or recurrence-free survival [52], symptoms of CIPN may result in patient reluctance to accept life-saving therapy and changes in treatment strategies, such as a reduction in chemotherapy dose intensity, changes in chemotherapy regimen, or even treatment discontinuance [9, 50].

Although it is recommended to prescribe duloxetine or pregabalin for CIPN in cancer patients, this clinical guideline does not work as a definite prevention or treatment modality [9, 13, 50]. Many studies have investigated safe, complementary treatments that have positive effects on CIPN symptoms, so invasive as well as non-invasive procedures were introduced. However, mixed results were obtained due to small sample sizes, an absence of placebo-controlled study designs, and evaluations done within a limited period [53]. This study has been designed to resolve the drawbacks of the previous studies. Specifically, a placebo control group is included in this study to ensure the effectiveness of the non-pharmacological intervention that was developed [20] because CIPN can disappear naturally by discontinuing chemotherapeutic agents that cause peripheral neuropathy [50]. Currently, there are no definite criteria or tools for evaluating CIPN [10, 37, 39]. In this study, various tools, such as the TNS [37], an instrument of pattern identification and evaluation for CIPN [46, 54], a numeral rating scale, FACT-B [55], and the EORTC QLQ-CIPN 20 questionnaire (the last item of the EORTC QLQ-CIPN 20 is omitted because of inappropriateness to female patients) will be administered to evaluate the symptoms of CIPN [6]. This study can contribute to a better understanding of CIPN-associated distress and its changing pattern or cancer-specific quality of life for individuals treated with adjuvant chemotherapy for breast cancer.

The electrostimulation device used in this study is non-invasive, portable, and easy to apply. In addition, this device is not dependent on the specific place that could be procedure or the ability of the operator. It can be applied anywhere or anytime and can be effective for a long duration without serious complications when compared to electronic acupuncture that only works for a short time [54]. All participants invited in both the experimental and sham-control groups will take either duloxetine or pregabalin as prescribed for their CIPN, and the electrostimulation device will be administered only to the experimental group. It is expected that the combination of a low-frequency electrostimulation device and pharmacological intervention (duloxetine or pregabalin) will produce synergistic effects in individuals with CIPN after breast cancer chemotherapy.

In this study, we set a follow-up period of 2 weeks. Previously, we reported one case of electroacupuncture for the treatment of CIPN in breast cancer patient. The patient was treated with Careband R for 14 days, in the same clinical settings as our present study. The symptoms of CIPN, measured by the Patient Neurotoxicity Questionnaire (PNQ) and visual analogue scale (VAS), significantly improved in this case [22]. Various clinical studies [20, 23–27] have reported the effect of scrambler therapy on CIPN. The treatment period of scrambler therapy was 10 days to 2 weeks. The results showed significant reduction in score of CIPN-related symptoms, which was measured by NRS at the end of the treatment period. Also several studies reported the long-term effect of scrambler therapy on CIPN, which was maintained to each follow-up period, at 5 weeks [23], 10 weeks [24], and 3 months [27].

The purposes of this study are to investigate the effect of low-frequency electrostimulation on CIPN symptoms and HRQOL of female patients diagnosed with CIPN immediately after chemotherapy for breast cancer. The findings of this study can provide healthcare professionals with valuable information on CIPN that can be considered during breast cancer treatment. Moreover, the results can be used to educate breast cancer patients and survivors to improve their abilities in monitoring and managing chemotherapy-related symptoms.

### Trial status

Patient recruitment is ongoing at the time of submission.

## Additional file

Additional file 1: Standard Protocol Items: Recommendations for Interventional Trials (SPIRIT) 2013 Checklist: recommended items to address in a clinical trial protocol and related documents. (DOC 121 kb)

## Abbreviations

CIPN: Chemotherapy-induced peripheral neuropathy
CIPN20: Chemotherapy-Induced Peripheral Neuropathy 20
ECOG: Eastern Cooperative Oncology Group
EORTC-QLQ: European Organization for Research and Treatment of Cancer Quality of Life
FACT-B: Functional Assessment of Cancer Therapy-Breast
HRQOL: Health-related quality of life
TENS: Transcutaneous electric nerve stimulation
TNS: Total Neuropathy Score

## References

[1] Kim Z, Min SY, Yoon CS (2015). The basic facts of Korean breast cancer in 2012: results from a nationwide survey and breast cancer registry database. J Breast Cancer.

[2] Oh CM, Won YJ, Jung KW (2016). Cancer statistics in Korea: incidence, mortality, survival, and prevalence in 2013. Cancer Res Treat.

[3] Korean Breast Cancer Society (2017). Breast Cancer Facts & Figures 2017.

[4] Howard-Anderson J, Ganz PA, Bower JE, Stanton AL (2012). Quality of life, fertility concerns, and behavioral health outcomes in younger breast cancer survivors: a systematic review. J Natl Cancer Inst.

[5] Pandey M, Thomas BC, SreeRekha P (2005). Quality of life determinants in women with breast cancer undergoing treatment with curative intent. World J Surg Oncol.

[6] Avis NE, Crawford S, Manuel J (2005). Quality of life among younger women with breast cancer. J Clin Oncol.

[7] Smith EM, Pang H, Cirrincione C (2013). Effect of duloxetine on pain, function, and quality of life among patients with chemotherapy-induced painful peripheral neuropathy: a randomized clinical trial. JAMA.

[8] Postma TJ, Aaronson NK, Heimans JJ (2005). The development of an EORTC quality of life questionnaire to assess chemotherapy-induced peripheral neuropathy: the QLQ-CIPN20. Eur J Cancer.

[9] Verstappen CC, Heimans JJ, Hoekman K, Postma TJ (2003). Neurotoxic complications of chemotherapy in patients with cancer: clinical signs and optimal management. Drugs.

[10] Seretny M, Currie GL, Sena ES (2014). Incidence, prevalence, and predictors of chemotherapy-induced peripheral neuropathy: a systematic review and meta-analysis. Pain.

[11] Miltenburg NC, Boogerd W (2014). Chemotherapy-induced neuropathy: a comprehensive survey. Cancer Treat Rev.

[12] Kukkar A, Bali A, Singh N, Jaggi AS (2013). Implications and mechanism of action of gabapentin in neuropathic pain. Arch Pharm Res.

[13] Hershman DL, Lacchetti C, Dworkin RH (2014). Prevention and management of chemotherapy-induced peripheral neuropathy in survivors of adult cancers: American Society of Clinical Oncology clinical practice guideline. J Clin Oncol.

[14] Rao RD, Michalak JC, Sloan JA (2007). Efficacy of gabapentin in the management of chemotherapy-induced peripheral neuropathy: a phase 3 randomized, double-blind, placebo-controlled, crossover trial (N00C3). Cancer.

[15] Moore RA, Wiffen PJ, Derry S, McQuay HJ. Gabapentin for chronic neuropathic pain and fibromyalgia in adults. Cochrane Database Syst Rev. 2011; 10.1002/14651858. 10.1002/14651858.CD007938.pub2 PMC4171034 21412914

[16] Franconi G, Manni L, Schroder S, Marchetti P, Robinson N (2013). A systematic review of experimental and clinical acupuncture in chemotherapy-induced peripheral neuropathy. Evid Based Complement Alternat Med.

[17] Bandla A, Sundar R, Liao LD (2016). Hypothermia for preventing chemotherapy-induced neuropathy - a pilot study on safety and tolerability in healthy controls. Acta Oncol.

[18] Garcia MK, McQuade J, Haddad R (2013). Systematic review of acupuncture in cancer care: a synthesis of the evidence. J Clin Oncol.

[19] Wong R, Major P, Sagar S (2016). Phase 2 study of acupuncture-like transcutaneous nerve stimulation for chemotherapy-induced peripheral neuropathy. Integr Cancer Ther.

[20] Smith TJ, Coyne PJ, Parker GL, Dodson P, Ramakrishnan V (2010). Pilot trial of a patient-specific cutaneous electrostimulation device (MC5-A Calmare(R)) for chemotherapy-induced peripheral neuropathy. J Pain Symptom Manag.

[21] Zhang R, Lao L, Ren K, Berman BM (2014). Mechanisms of acupuncture-electroacupuncture on persistent pain. Anesthesiology.

[22] Ji Hye Park JSL, Cho CK, Yoo HS (2015). Electroacupuncture for the treatment of the chemotherapy-induced peripheral neuropathy in breast cancer patient: a case report. J Korean Tradit Oncol.

[23] Deirdre R, Breanna M, Debra L, et al. Pilot study of scrambler therapy for the treatment of chemotherapy-induced peripheral neuropathy. J Clin Oncol. 2012;30(15_Suppl):9075–9075. http://ascopubs.org/doi/abs/10.1200/jco.2012.30.15_suppl.9075. 10.1200/jco.2012.30.15_suppl.9075.

[24] Breanna L, Deirdre R, Drew K (2014). Scrambler therapy for treatment of chemotherapy-induced peripheral neuropathy. J Clin Oncol.

[25] Park HS, Sin WK, Kim HY (2013). Scrambler therapy for patients with Cancer pain—case series. Korean J Pain.

[26] Deirdre R (2015). Pilot evaluation of scrambler therapy for the treatment of chemotherapy-induced peripheral neuropathy. Support Care Cancer.

[27] Coyne PJ, Wan W, Dodson P, Swainey C, Smith TJ (2013). A trial of scrambler therapy in the treatment of cancer pain syndromes and chronic chemotherapy-induced peripheral neuropathy. J Pain Palliat Care Pharmacother.

[28] Wolf Sherry L., Barton Debra L., Qin Rui, Wos Edward J., Sloan Jeff A., Liu Heshan, Aaronson Neil K., Satele Daniel V., Mattar Bassam I., Green Nathan B., Loprinzi Charles L. (2011). The relationship between numbness, tingling, and shooting/burning pain in patients with chemotherapy-induced peripheral neuropathy (CIPN) as measured by the EORTC QLQ-CIPN20 instrument, N06CA. Supportive Care in Cancer.

[29] Ricci M, Pirotti S, Scarpi E (2012). Managing chronic pain: results from an open-label study using MC5-A Calmare(R) device. Support Care Cancer.

[30] Feeley N, Cossette S, Cote J (2009). The importance of piloting an RCT intervention. Can J Nurs Res.

[31] Hertzog MA (2008). Considerations in determining sample size for pilot studies. Res Nurs Health.

[32] Hammes M, Kuschick N, Christoph KH. Handbook of acpuncture. 1st ed. Seoul: Hansol Medical Book Co Ltd; 2010.

[33] Paice JA, Cohen FL (1997). Validity of a verbally administered numeric rating scale to measure cancer pain intensity. Cancer Nurs.

[34] Jensen MP (2003). The validity and reliability of pain measures in adults with cancer. J Pain.

[35] Cornblath DR, Chaudhry V, Carter K (1999). Total Neuropathy Score: validation and reliability study. Neurology.

[36] Cavaletti G, Bogliun G, Marzorati L (2003). Grading of chemotherapy-induced peripheral neurotoxicity using the Total Neuropathy Scale. Neurology.

[37] Cavaletti G, Frigeni B, Lanzani F (2007). The Total Neuropathy Score as an assessment tool for grading the course of chemotherapy-induced peripheral neurotoxicity: comparison with the National Cancer Institute-Common Toxicity Scale. J Peripher Nerv Syst.

[38] Park SB, Goldstein D, Krishnan AV (2013). Chemotherapy-induced peripheral neurotoxicity: a critical analysis. CA Cancer J Clin.

[39] Cavaletti G, Jann S, Pace A (2006). Multi-center assessment of the Total Neuropathy Score for chemotherapy-induced peripheral neurotoxicity. J Peripher Nerv Syst.

[40] Cella DF, Tulsky DS, Gray G (1993). The Functional Assessment of Cancer Therapy scale: development and validation of the general measure. J Clin Oncol.

[41] Brady MJ, Cella DF, Mo F (1997). Reliability and validity of the Functional Assessment of Cancer Therapy-Breast quality-of-life instrument. J Clin Oncol.

[42] Ng R, Lee CF, Wong NS (2012). Measurement properties of the English and Chinese versions of the Functional Assessment of Cancer Therapy-Breast (FACT-B) in Asian breast cancer patients. Breast Cancer Res Treat.

[43] Yoo HJ, Ahn SH, Eremenco S (2005). Korean translation and validation of the functional assessment of cancer therapy-breast (FACT-B) scale version 4. Qual Life Res.

[44] Kim HY, Kang JH, Youn HJ (2014). Reliability and validity of the Korean version of the European Organization for Research and Treatment of Cancer Quality of Life Questionnaire to assess chemotherapy-induced peripheral neuropathy. J Korean Acad Nurs.

[45] Lavoie Smith EM, Barton DL, Qin R, Steen PD, Aaronson NK, Loprinzi CL (2013). Assessing patient-reported peripheral neuropathy: the reliability and validity of the European Organization for Research and Treatment of Cancer QLQ-CIPN20 questionnaire. Qual Life Res.

[46] Ji-hye Park I-cJ, Lee S-h, Lee J-s, Bae K-r, Cho C-k, Yoo H-s (2016). Preliminary study to develop an instrument for pattern identification and evaluation for chemotherapy-induced peripheral neuropathy (CIPN). J Korean Orient Intern Med.

[47] Murphy BA (2009). Advances in quality of life and symptom management for head and neck cancer patients. Curr Opin Oncol.

[48] Irvin W, Muss HB, Mayer DK (2011). Symptom management in metastatic breast cancer. Oncologist.

[49] Carlson K, Ocean AJ (2011). Peripheral neuropathy with microtubule-targeting agents: occurrence and management approach. Clin Breast Can.

[50] Manji H (2011). Toxic neuropathy. Curr Opin Neurol.

[51] Rivera DR, Ganz PA, Weyrich MS, Bandos H, Melnikow J. Chemotherapy-associated peripheral neuropathy in patients with early-stage breast cancer: a systematic review. J Natl Cancer Inst. 2018;110. 10.1093/jnci/djx140. 10.1093/jnci/djx140 PMC5825681 28954296

[52] Schneider BP, Zhao F, Wang M (2012). Neuropathy is not associated with clinical outcomes in patients receiving adjuvant taxane-containing therapy for operable breast cancer. J Clin Oncol.

[53] Hsieh YL, Chou LW, Hong SF (2016). Laser acupuncture attenuates oxaliplatin-induced peripheral neuropathy in patients with gastrointestinal cancer: a pilot prospective cohort study. Acupunct Med.

[54] Choi JW, Kang SY, Choi JG (2015). Analgesic effect of electroacupuncture on paclitaxel-induced neuropathic pain via spinal opioidergic and adrenergic mechanisms in mice. Am J Chinese Med.

[55] Park J-h, Jung I-c, Lee S-h, Lee S-h, Choi S-c, Yoo H-s (2016). Reliability and validity analysis of an instrument for pattern identification and evaluation in chemotherapy-induced peripheral neuropathy. J Intern Korean Med.

